# From inflammation to bone formation: the intricate role of neutrophils in skeletal muscle injury and traumatic heterotopic ossification

**DOI:** 10.1038/s12276-024-01270-7

**Published:** 2024-07-01

**Authors:** Lin-Zhen Shu, Xian-Lei Zhang, Yi-Dan Ding, Hui Lin

**Affiliations:** 1 School of Basic Medical Sciences, Jiangxi Medical College, Nanchang University, 330006 Nanchang, Jiangxi China; 2 Medical College, Nanchang University, 330006 Nanchang, Jiangxi China

**Keywords:** Cell biology, Immunology

## Abstract

Neutrophils are emerging as an important player in skeletal muscle injury and repair. Neutrophils accumulate in injured tissue, thus releasing inflammatory factors, proteases and neutrophil extracellular traps (NETs) to clear muscle debris and pathogens when skeletal muscle is damaged. During the process of muscle repair, neutrophils can promote self-renewal and angiogenesis in satellite cells. When neutrophils are abnormally overactivated, neutrophils cause collagen deposition, functional impairment of satellite cells, and damage to the skeletal muscle vascular endothelium. Heterotopic ossification (HO) refers to abnormal bone formation in soft tissue. Skeletal muscle injury is one of the main causes of traumatic HO (tHO). Neutrophils play a pivotal role in activating BMPs and TGF-β signals, thus promoting the differentiation of mesenchymal stem cells and progenitor cells into osteoblasts or osteoclasts to facilitate HO. Furthermore, NETs are specifically localized at the site of HO, thereby accelerating the formation of HO. Additionally, the overactivation of neutrophils contributes to the disruption of immune homeostasis to trigger HO. An understanding of the diverse roles of neutrophils will not only provide more information on the pathogenesis of skeletal muscle injury for repair and HO but also provides a foundation for the development of more efficacious treatment modalities for HO.

## Neutrophils in Muscle Injury: Implications for Heterotopic Ossification

Skeletal muscle, the body’s most common tissue, often gets injured and lacks highly effective treatments. This review investigates the complex relationship between skeletal muscle and neutrophils during injury and healing. Researchers study how neutrophils can both worsen muscle damage and assist in tissue repair. It looks at how neutrophil activity affects muscle repair, explaining the processes of inflammation, tissue regeneration, and the factors causing heterotopic ossification. It also emphasizes the significance of controlling neutrophil activity for effective muscle healing and avoiding complications. Key findings show that neutrophils are crucial in both harming and repairing skeletal muscle. Overactive neutrophils can cause extended inflammation, hindering the healing process, while controlled activity aids tissue regeneration. The researchers suggest that focusing on neutrophil activity could be a promising method for treating muscle injuries and preventing heterotopic ossification.

This summary was initially drafted using artificial intelligence, then revised and fact-checked by the author.

## Introduction

Skeletal muscle, which is also known as striated muscle, is the most abundant tissue in the human body^1,2^. Skeletal muscle injury is frequently clinically caused by trauma, ischemia-reperfusion, burns, and even physical exercise but has not been linked to a high-efficiency treatment until now^2^. Neutrophils are the first infiltrating immune cells in damaged tissue and play diverse roles in the inflammatory response^3^. Neutrophils are the first type of cell to defend against invading infections caused by microorganisms; moreover, they are also essential for regulating the process of tissue repair and regeneration^4^. Over the past few years, an increasing number of studies have focused on the complex crosstalk between skeletal muscle and neutrophils in acute injuries and chronic diseases^1^. Infiltrated neutrophils exacerbate the original damage by assisting in the removal of damaged tissue and the release of free radicals and protein hydrolases^1^. Thus, overactive neutrophils can be significantly destructive and can lead to severe tissue injury; moreover, they can exacerbate skeletal muscle damage. Interestingly, neutrophils have also been shown to contribute to tissue repair in different organs, including the liver, lung, and bone^5^. Different subtypes of neutrophils may have different functions during tissue repair, and more efficient phagocytosis or cytokine production and dysregulation of neutrophil heterogeneity may lead to impaired wound healing^6^.

Heterotopic ossification (HO) is defined as the formation of bone in soft tissues and joints^7^. HOs are broadly divided into traumatic HO (tHO), neurogenic HO (NHO), and genetic HO. It is commonly recognized as being a complication after trauma, surgery, spinal cord injury (SCI), or other injuries. Patients with HO suffer from joint ankylosis, which is difficult to treat, and muscle pain. tHO is most commonly induced by tissue injury, followed by inflammation and subsequent events, which inappropriately activate osteogenic or osteochondrogenic mechanisms^8^. Inflammatory factors recruit immune cells to create a microenvironment that serves as a vital “niche” in HO initiation and progression. A heterogeneous population of cells responds to immune cells recruited by inflammatory osteoplastic signals to initiate the bone formation process^9^. Muscle injury and its associated inflammation appear to be indispensable triggers for HO^10^; however, the injury-induced osteogenic mechanism has not yet been identified. The role of macrophages in the HO process has been explored in detail^9^. However, as neutrophils are the earliest recruited immune cells in injured tissues, the role of neutrophils in the HO-associated inflammatory process remains unclear, and neutrophils are considered to be able to recruit macrophages. Neutrophils and neutrophil extracellular traps (NETs) reportedly promote osteogenesis in skeletal muscle injury and play a role in the HO process^11^. Moreover, there is a crosstalk between neutrophils and macrophages, as well as nerve signaling, which represents the major drivers of HO.

Herein, we review the molecular roles of neutrophils in skeletal muscle injury, repair, and the process of HO. The elucidation of the vital role of neutrophils provides a theoretical basis for treatment by targeting neutrophils in skeletal muscle injury-related diseases and HO.

## Neutrophils in skeletal muscle injury, repair and regeneration

Neutrophils, which originate from the bone marrow, constitute approximately 60–70% of the total leukocyte population^3,12^. In the classical view, neutrophils are generated from hematopoietic stem cells and differentiate into subpopulations of neutrophil killers and neutrophil capsers^13,14^. Granulocyte colony-stimulating factor (G-CSF) is a critical regulator of neutrophil expansion and mobilization from the bone marrow^15^. Furthermore, neutrophil recruitment to sites of inflammation also affects the overall frequency of neutrophils^16^. When they are generated in the bone marrow, neutrophils survive for only 6–8 h in circulation, after which they are cleared^13,17^. Neutrophils play one of the most well-known roles in inflammatory and immune processes and are also extremely diverse. Neutrophils are effective antimicrobial cells and represent the first line of cellular defense against infection by deploying antimicrobial elements, thus resulting in tissue damage^12^. After being recognized, neutrophils activate multiple antimicrobial mechanisms, including phagocytosis of cellular debris; degranulation of antimicrobial enzymes, such as neutrophil elastase (NE) and myeloperoxidase (MPO); DNA webs; and NETs^1,17^. The role of neutrophils in tissue injury and regeneration, in addition to infectious diseases, is becoming better understood^18–20^. For example, neutrophils can significantly enhance angiogenesis to promote the healing of fractures^21^. In conclusion, during their short life, the phenotype of neutrophils substantially changes with changes in the environment.

Skeletal muscle is physiologically important to humans and suffers more damage than other human tissues under normal conditions^22^. Skeletal muscle injuries can be divided into acute and chronic injuries. Acute injuries are caused by perturbations applied over a short period of time, such as lacerations, contusions, frigidity, burns, or exposure to toxins. In injured skeletal muscle, pathogen-associated molecular pattern (PAMP) and damage-associated molecular pattern (DAMP) signals are released, and various immune cells are recruited^23^. After skeletal muscle injury, the activated complement system and leaky muscle cells recruit neutrophils to participate in anti-infection reactions^17,24^. Neutrophils first remove tissue fragments and microorganisms from necrotic and atrophic skeletal muscle, which is beneficial for subsequent skeletal muscle repair. Moreover, neutrophil-derived cytokines, proteolytic enzymes, and NETs are also involved in this process^25^.

**Fig. 1:**
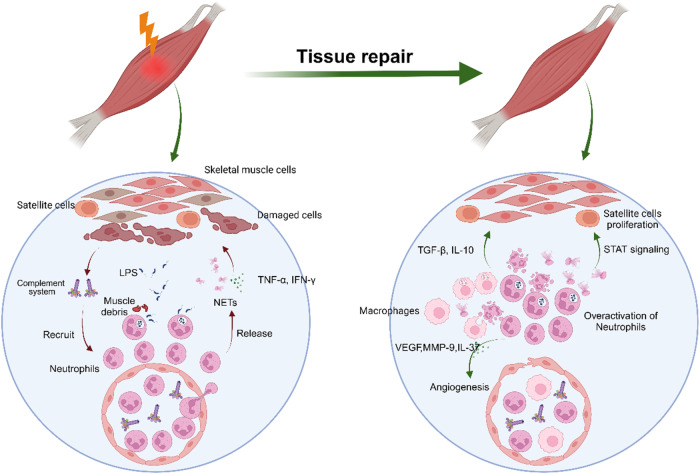
Role of neutrophils in skeletal muscle repair. Neutrophils are mobilized by complement to migrate toward injured skeletal muscle tissue to eliminate LPS and muscle debris at the injury site. Following their recruitment, neutrophils release TNF-α, IFN-γ, and NETs to affect damaged skeletal muscle. In the repair phase, neutrophils can enhance the renewal and proliferation of satellite cells through the STAT signaling pathway, thereby contributing to the regeneration of damaged muscle tissue. Moreover, neutrophils release VEGF, MMP-9, and IL-37 to promote angiogenesis to provide nutrients that are necessary for tissue regeneration. After neutrophil apoptosis, macrophages engulf apoptotic remnants, thus triggering the secretion of TGF-β and IL-10, which act on skeletal muscle to restore normal contraction and relaxation.

The repair of skeletal muscle involves inflammatory responses, muscle fiber regeneration, angiogenesis, and extracellular matrix (ECM) remodeling^26^. Infiltrated neutrophils release vascular endothelial growth factor (VEGF), and matrix metalloproteinase-9 (MMP-9) after the MAPK signaling pathway is activated^25,27–29^, thus promoting angiogenesis in injured muscle tissue. Moreover, neutrophils produce secretory leukocyte protease inhibitors (SLPIs) to accelerate wound healing after the NF-κB signaling pathway is activated^30^. Satellite cells mediate the initiation of muscle regeneration, regenerate damaged muscle fibers, and restore muscle contractility and metabolism^26^. In response to trauma, satellite cells are activated to enter the cell cycle, proliferate briefly, and further differentiate into new muscle tubes or fuse with damaged muscle fibers to repair muscle injury. The infiltration of neutrophils is also beneficial for muscle regeneration through the activation of satellite cells via the STAT pathway^31–34^. At the later stage of skeletal muscle repair, neutrophils gradually undergo apoptosis or migration, and apoptotic neutrophils are engulfed by macrophages, which initiates a feedback repair program and releases the tissue repair factors TGF-β and IL-10 through the TGF-β/Smad2 signaling pathway and AKT/GSK3β pathway, respectively. This subsequently promotes the remodeling of the ECM of skeletal muscle and is beneficial for restoring the normal contraction and relaxation function of skeletal muscle^35^ (Fig. 1).

**Fig. 2:**
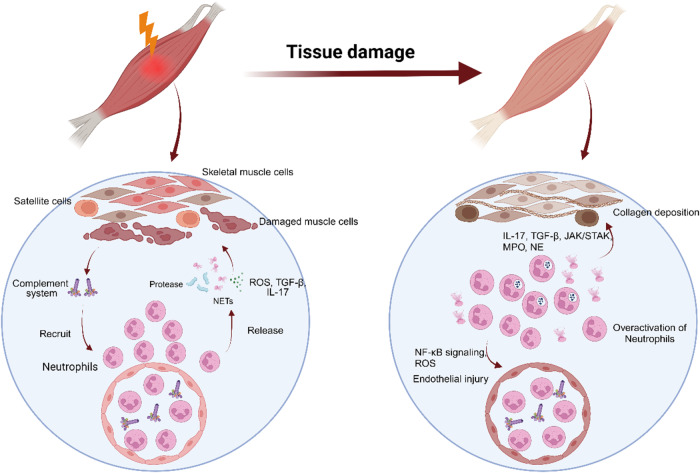
Role of neutrophils in skeletal muscle injury. Following skeletal muscle injury, neutrophils release ROS, NETs, TGF-β, IL-17, and proteases through complement recruitment to the injured site. However, the overactivation of neutrophils can result in continuous infiltration of neutrophils and the formation of NETs at the injured site. Collagen deposition and impaired regeneration of satellite cells occur through the release of IL-17, TGF-β, MPO, NE, and JAK/STAT signals. Furthermore, the excessive release of ROS and activation of NF-κB in neutrophils can directly damage the vascular endothelium in muscle tissue, thus hindering muscle repair and regeneration and eventually causing muscle atrophy.

However, the overactivation of neutrophils, which results in persistent inflammatory activation, leads to tissue damage. Continuous inflammatory reactions inhibit the repair of injured skeletal muscle and cause muscle atrophy^2^. Afterward, the overreaction affects the release of superoxide anion radicals and/or hydrogen peroxide by neutrophils, which leads to further tissue damage^36^. After skeletal muscle atrophy or necrosis after injury, due to the deposition of fibrinogen, vascular permeability in diseased skeletal muscle increases, which accelerates the process of skeletal muscle repair^33^. However, fibrinogen also promotes the recruitment of neutrophils through interactions with the macrophage-1-antigen integrin receptor, which may lead to further overactivation of neutrophils and aggravate skeletal muscle injury^37^. Reactive oxygen species (ROS) released by neutrophils through the NF-κB signaling pathway are beneficial for muscle fiber degeneration and vascular changes caused by ischemia-reperfusion injury^36,38^. However, excessive ROS damages the vascular endothelium in muscle tissue to hinder the regeneration process of muscle^39,40^. Overactive neutrophils secrete IL-17 and TGF-β, which leads to the activation of stellate cells and the proliferation of adipose and connective tissue, thus ultimately limiting the function of repaired skeletal muscle^41–45^. The persistent infiltration of neutrophils in degenerative volumetric muscle loss injuries has been shown to contribute to reducing the number of muscle satellite cells that are needed for muscle cell regeneration, which was also attenuated by the JAK/STAK signaling pathway^46^. NETs also play a very special role in the damage of neutrophils to skeletal muscle. It is generally believed that NETs can reverse inflammation by degrading cytokines and chemokines and hinder the excessive recruitment of inflammatory cells^47^. However, excessive NETs can lead to tissue injury. In the presence of NETs, MMPs are released through the MAPK signaling pathway, thus leading to skeletal muscle reinjury^48^. MPO and NE promote the prolongation of inflammatory reactions, lead to oxidative stress, and delay the repair of skeletal muscle injury^17^ (Fig. 2).

## Skeletal muscle injury to HO

HO requires inducible bone stem or progenitor cells and an ectopic environment conducive to osteogenesis^49^. Burns and mechanical injuries are major contributors to trauma-induced skeletal muscle damage and may further cause tHO^50^. During skeletal muscle injury, the first recruited immune cells are T-helper cells and neutrophils, after which macrophages dominate infiltration after approximately 24 h^51,52^. Neutrophils secrete TNF-α, IL-1β, and IL-1α, thus creating an inflammatory microenvironment^53–56^. This represents the initial phase of muscle repair, followed by two subsequent phases of muscle regeneration and fibrosis, with muscle repair being a delicate equilibrium between muscle regeneration and fibrosis. Nevertheless, the overactivation of neutrophils may heighten the probability of scarring or fibrosis by aggravating muscle damage, which is the primary factor contributing to the gradual decline in muscle functionality and the occurrence of HO^57–59^. Thus, the appropriate presence and activation of neutrophils are crucial for the management of muscle injury and HO.

Neutrophil-derived TGF-β and BMP signaling drive osteogenic differentiation. TGF-β signaling regulates scleraxis expression in skeletal muscle fibroblasts, thereby facilitating proliferation and collagen type-I synthesis, both of which are critical for effective tissue repair^60^. Furthermore, BMP signaling leads to the differentiation of MSCs toward the osteogenic lineage^61^. This mechanism is a potential method of triggering tHO in vivo, particularly when neutrophils are overactivated and release BMP in conjunction with TGF-β. Furthermore, TGF-β can bind to Smad2/3, which is activated to regulate inflammation when ActA is combined with BMP receptors, thus suggesting that BMP associates with TGF-β to induce strong tHO^62,63^. Smad1/5/8 can be phosphorylated and translocated to the nucleus in the presence of BMP-2 signaling alone in C2C12 cells, where it converts C2C12 cells to osteoblasts^61^. Interestingly, TGF-β inhibits HO by reducing the nuclear translocation of the downstream protein Smad1/5/8 and preventing activation of the Wnt pathway^64^. Janna et al. used metabolic profiling to demonstrate that itaconate, which is produced by mature neutrophils at injury sites, is a metabolite that is differentially abundant between HO and non-HO in burn/tenotomy models of the Achilles tendon^65^. These mature neutrophils contribute to prolonged inflammation by secreting the cytokines CCL2 and IL-1β. Moreover, itaconate-producing neutrophils return to the bone marrow for degradation by macrophages and turnover of hematopoiesis to the myeloid lineage.

Trauma can also lead to a local and systemic inflammatory state with elevated inflammatory cytokines, such as TNFα, IL-1β, and IL-6^66^. The persistence of the inflammatory microenvironment is a significant alteration in the development of HO, thus allowing for neutrophils and macrophages to be overactivated and further leading to abnormal osteogenic differentiation of MSCs by affecting the balance of bone formation and resorption through the NF-κB signaling pathway, thereby promoting HO^66,67^.

**Fig. 3:**
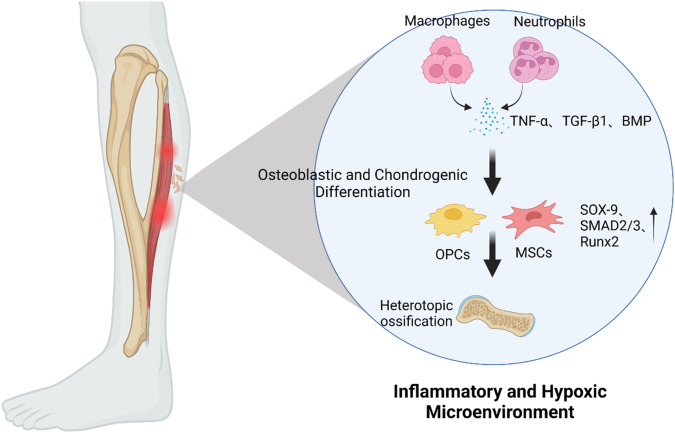
Progression of skeletal muscle injury to heterotopic ossification. Following skeletal muscle injury, the infiltration of neutrophils and macrophages results in a localized inflammatory and hypoxic microenvironment. These neutrophils and macrophages consistently release cytokines, including TNF-α, TGF-β1, and BMP, which facilitate the differentiation of MSCs and OPCs (markers) into osteoblasts and chondrocytes by upregulating SOX-9, SMAD2/3, Runx2, and other factors. This process culminates in the development of ectopic ossification.

Overactivation of neutrophils causes a traumatic site to develop into a hypoxic environment^68,69^. Additionally, neutrophils upregulate the transcription factor SOX-9, thereby promoting chondrocyte differentiation^70,71^. Consequently, chondrocytes undergo excessive proliferation and initiate the formation of a cartilage matrix, thus ultimately leading to the occurrence of HO^72–74^. Furthermore, hypoxia-inducible factor (HIF) serves as a regulator of BMP-2-induced endochondral osteogenesis in fetal limb culture^75^. Significantly, the synergistic relationship between HIF and VEGF amplifies the expression of endothelial osteogenic growth factors, thereby facilitating osteoblast differentiation but also attracting bone-forming stem cells to the injured tissue^72^ (Fig. 3).

As shown above, during the repair process of trauma-induced skeletal muscle injury, complex interactions among inflammatory cells, neutrophils, macrophages, and a hypoxic environment are the main causes of tHO. Importantly, compared with those in the blood circulation or bone marrow, neutrophils at the site of HO strongly express inflammatory cytokines to prolong local inflammation and support abnormal cell differentiation. This finding implies that there are local factors at the injury site that mediate the differences in neutrophils. Moreover, macrophages are extremely versatile cells, and the function of these cells mainly depends on the local environment. HO site neutrophils homed to the bone marrow and promoted the myeloid differentiation of bone marrow stem cells. Locally present neutrophils alter the phenotype of subsequently recruited macrophages, thus making them a major contributor to subsequent HO progression. Although macrophages and neutrophils are capable of producing cytokines, the mechanism of how they are abnormally activated by trauma and how they produce sufficient amounts of cytokines (such as BMP and TGF-β) to cause tHO in vivo remain open questions.

## Molecular mechanism of neutrophils in tHO

tHO requires three osteogenic conditions: osteogenic signal induction factors, osteogenic precursor cells, and an appropriate local microenvironment^76,77^. BMP signaling is one of the most common osteogenic signaling pathways for HO-forming progenitor cells^78^. Neutrophils significantly regulate major osteogenic markers, such as BMP 2-5, TGF-β2, RUNX2, and extracellular matrix (ECM) proteins^79^. Moreover, neutrophils activate MSCs and cause osteogenic differentiation accompanied by increased levels of IL-1α and TGF-β^80^. The neutrophil-mediated inflammatory response enhances the chondrocyte differentiation of MSCs^81^. Cai et al. demonstrated that neutrophils are initially recruited following IL-8 implantation and transition to the N2 type^82^. Subsequently, N2-neutrophil-derived stromal cell-derived factor-1α (SDF-1a) migrates through the SDF-1/CXCR4 axis, thus leading to PI3K/Akt- and β-catenin-mediated migration of bone MSCs for neutrophil-initiated ossification. The depletion of neutrophils can hinder the recruitment of all bone MSCs. N2-neutrophils also play a role in inducing an anti-inflammatory phenotypic transformation in macrophages. Through single-cell RNA-seq, Nunez et al. ascertained that NETs were not present in the bone marrow or blood subsequent to injury but were rather manifested solely at the site of musculoskeletal injury^11^. Beginning on Day 3, the formation of NETs at the musculoskeletal injury site was notably greater in the HO model group than in the normal repair group on Day 7 following burn and skin incision injury. Furthermore, this localized NET formation corresponded with an elevated level of neutrophil-TLR signaling at the injury site^11^.

Vascular smooth muscle cells and pericellular nerve growth factor (NGF), which mediate tropomyosin receptor kinase A (TrkA)^+^ nerve intrusion into soft tissue trauma sites, are key features in the pathogenesis of tHO^83–85^. After a tendon injury, neurogenesis occurs near NGF^+^ tendon cells, and the destruction of sensory innervation or inhibition of NGF-TrkA signaling leads to an imbalance of inflammatory and TGF-β signaling in injured mouse tendons, thus limiting tendon repair^86^. NGF-p75 signaling is also activated after skull injury, and NGF-p75 recruits resident mesenchymal osteogenic precursors for migration to damaged tissue^87^. However, the overexcitability of NGF-TrkA signaling leads to the excessive innervation of nerves at the injured site, thus resulting in the active expression of cartilage antigens in the injured tendon and the transformation of FGF to TGF-β signaling, which promotes the progression of ectopic bone^84,85^. Neutrophils can produce NGF and are involved in nerve regeneration. Neutrophils can be used for neuroprotection and axon generation through NGF and IGF-1 signaling, thus suggesting that neutrophils can promote HO progression through NGF signaling^88,89^. It is possible that in the early stages of injury, NGF^+^ neutrophils are recruited for neuroprotection. However, tissue injury triggers smooth muscle cells to release abnormal NGF signals into the local inflammatory microenvironment, which recruits TrkA^+^ neutrophils to promote neutrophil trapping and the promotion of the progression of HO^84^.

Neutrophils are also able to influence osteoblasts and osteoclasts through Il-1β, Il-6, IFN-γ, and TNF-α^90^. Furthermore, the presence of neutrophils eliminates pathogenic microorganisms in a timely manner, thus potentially creating conditions conducive to HO. Davisbk et al. demonstrated that macrophages stimulated by LPS are more prone to convert to M1 macrophages through the IRF/STAT signaling pathway, which has the potential to become osteoclasts^91^. However, overactivated neutrophils convert macrophages to M2 macrophages, which triggers osteogenesis via MSCs^82,91^.

**Fig. 4:**
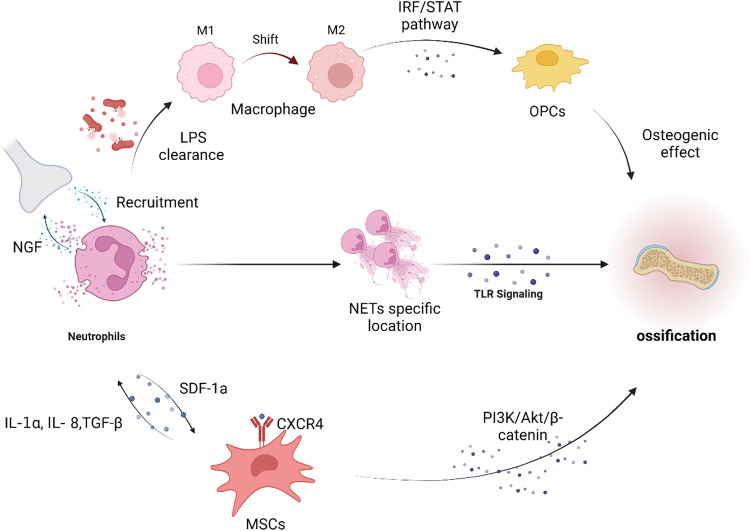
Role of neutrophils in heterotopic ossification. The overactivation of neutrophils can contribute to the occurrence of HO. Neutrophils interact with MSCs via an IL-Lα, IL-8, and TNF-α loop and release SDF-1α to stimulate the chondrocyte differentiation of MSCs through the CXCR4-PI3K/Akt/β-catenin pathway. Overactivation of neutrophils also leads to significant local infiltration of NETs, which promotes TLR9 signaling and directly facilitates the formation of HO. Furthermore, neutrophils directly eliminate LPS, thereby creating an environment conducive to the shift of macrophages from the M1 to the M2 osteogenic phenotype, which subsequently enhances the development of HO.

The mechanisms by which neutrophils interact with other cells in the microenvironment, such as osteoblasts and osteoclasts, are necessary to elicit HO and still require further investigation. Additionally, when considering the limited lifespan of neutrophils, the development of in vitro models and enhanced culture methods for studying neutrophils may contribute to a more comprehensive understanding of their involvement in the formation of HO (Fig. 4).

## Targeting neutrophils in skeletal muscle injury and HO therapies

**Fig. 5:**
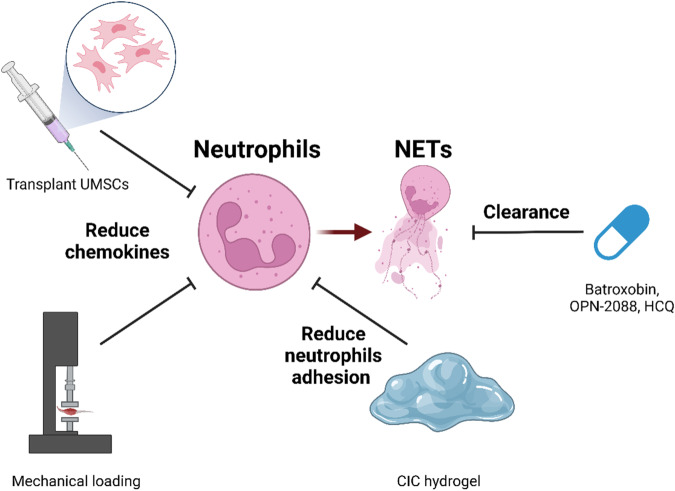
Targeting neutrophils to skeletal muscle injury and heterotopic ossification. The treatment of skeletal muscle injury and HO is achieved by targeting neutrophils and the resulting NETs in the injured microenvironment. Through targeted reduction of neutrophil chemokines, the transplantation of umbilical cord MSCs (UMSCs) and mechanical loading can reduce the number of damaged neutrophils to reduce muscle injury. Moreover, by reducing the adhesion of neutrophils, a hydrogel known as CIC can be used to control injury-induced inflammation. In addition, batroxobin, hydroxychloroquine (HCQ), and the TLR9 inhibitor OPN-2088 can inhibit HO by clearing NETs.

Due to the critical and complex roles of neutrophils in skeletal muscle injury and repair in HO, we need to distinguish between secondary responses and potentially deleterious side effects. A better understanding of the roles of neutrophils may help us to consider them as promising therapeutic targets (Fig. 5).

Cyclic compressive loading within a specific range of forces directs the rapid clearance of neutrophils to improve muscle regeneration^92^. Another promising route for combating the inflammatory response caused by muscle injury is to transplant umbilical cord MSCs (UMSCs). Studies have shown that UMSCs can attenuate neutrophil-derived acute inflammation by ameliorating early-onset neutrophil infiltration and activation, thereby mitigating the extent of inflammation^53^. Some drugs have also been found to be effective in the treatment of muscle injury. A hydrogel system consisting of CD146, IGF-1, collagen I/III, and poloxamer 407 (termed CIC gel), which secretes CD146 to effectively promote efferocytosis and neutrophil adhesion, could be a promising tool for impeding muscle inflammation^93^. Similarly, a neutrophil-mediated delivery system loaded with liposomes was discovered to have the potential to deliver anti-inflammatory drugs such^94^ as methotrexate (a potent immunosuppressive agent used to treat inflammation) without impairing neutrophil viability and chemotaxis, through which neutrophils can retain their ability to rapidly migrate to inflamed tissue and subsequently release drug-loaded liposomes, thus causing the intended anti-inflammatory effect^94^. Nunez demonstrated that reducing the overall neutrophil abundance at the injury site also mitigated HO formation, whether through pharmacological treatment with hydroxychloroquine (HCQ), treatment with the TLR9 inhibitor OPN-2088, or mechanical treatment with limb unloading^11^. Several studies have demonstrated the ability of batroxobin to abrogate neutrophil extracellular traps, which may be used to suppress inflammation and expedite myoblast regeneration^95^. However, the mechanism by which neutrophils function in skeletal muscle injury leading to HO remains largely unknown, and more research is needed to fill in the knowledge gaps for neutrophils to be viable targets.

## Conclusion and future perspective

Neutrophils are the initial immune cells recruited at the site of skeletal muscle injury. They eliminate skeletal muscle debris and microorganisms through the release of inflammatory factors, protein hydrolases, and NETs. Following anti-infection measures, NETs facilitate the resolution of inflammation by degrading cytokines and chemokines, thus impeding the excessive recruitment of inflammatory cells. Neutrophils can generate VEGF and MMP-9 to promote tissue repair. Additionally, apoptotic neutrophils can stimulate macrophages, thus leading to the release of TGF-β and IL-10, thereby facilitating the remodeling of the extracellular matrix in skeletal muscle and aiding in the restoration of normal contractile and diastolic functions. However, neutrophil overactivation and the persistence of NETs can result in prolonged inflammation, which ultimately impairs the functionality of satellite cells and limits the overall repair process. Skeletal muscle injury is a prominent contributor to tHO. Neutrophil overactivation and the resulting immunological disturbances associated with skeletal muscle injury can significantly stimulate the production of NETs, as well as the activation of the TGF-β1 and BMP signaling pathways in local tissue. This dysfunctional inflammatory microenvironment serves as a catalyst for the osteogenic differentiation of various stem cells, including MSCs. Neutrophils can exert systemic effects on osteoblasts and osteoclasts through a systemic inflammatory response. Moreover, neutrophils also contribute to HO progression by affecting NGF signaling and myeloid hematopoietic bias. However, it is important to note that neutrophils play dual roles in the early stages of muscle injury, either by promoting muscle repair or contributing to the development of HO. In view of the great heterogeneity of circulating neutrophils and neutrophils at the site of HO, the local upstream signaling mediating this heterogeneity is worthy of investigation. In conclusion, neutrophils themselves may play a considerable role in the initiation and progression of HO, and it is likely that they are also capable of regulating the role of other cells and neural signaling. To date, studies of neutrophils in HO are still scarce, and the mechanisms underlying their interaction with other cells and secreted cytokines still need to be elucidated. For example, the mechanisms by which neutrophils are overactivated in the injured microenvironment and how NETs are specifically localized at the injury site remain unknown and need to be discussed in the future. This cell interaction may occur as early as in the bone marrow and is of great concern. In summary, neutrophils potentially exert a significant influence on the onset and progression of HO, and they may also possess the ability to modulate the functions of other immune cells. Currently, investigations pertaining to neutrophils in the context of HO are limited, and further elucidation is required regarding their interactions with other cells and the cytokines that they secrete, as well as for expanding the study to the whole body rather than just the injured site. Moreover, given the transient lifespan of neutrophils, enhancing in vitro models and culture techniques for these cells would positively contribute to a more comprehensive understanding of their role in HO.
